# News and Coming Events

**Published:** 1909-02-20

**Authors:** 


### News and Coming Events.

The Senatus Academicus of the University of Edinburgh has resolved to offer the honorary LL.D. degree to Sir Alfred Keogh, K.C.B., Director-General of the Army Medical Service.

Professor Dieudonne, who went to India in 1897 to investigate the plague epidemic as a member of Professor Koch's commission, has been appointed "Referent" for the kingdom of Bavaria in succession to Dr. von Grashey who is retiring.

The late Mr. J. A. D. Shipley, of Newcastle, left the whole of the residue of his estate, which is roughly estimated to be about £100,000, for division among various local charities, including the Royal Victoria Infirmary and other medical charities.

Mr. John Rowntree, shipowner, North Shields, who died on December 12 last, aged ninety-one years, bequeathed £1,000 to the Tynemouth Victoria Jubilee Infirmary, £250 to the North Shields Dispensary, and £250 to the Indigent Sick Society, North Shields.

The result of the January examination for commissions in the Indian Medical Service was announced on January 30. There were 38 candidates, of whom 33 ultimately entered for the examination; of these 31 qualified, the first 12 being admitted as lieutenants-on-probation, while one withdrew during the examination.

The death occurred suddenly, on February 13, of Dr. John Tawse Nisbet, of Liverpool. While attending a patient he was seized with a sudden attack of syncope, and died at the patient's bedside. Dr. Nisbet graduated at Edinburgh University, and started practice in Liverpool in 1884. For fifteen years he was assistant physician to the Liverpool Children's Infirmary. He was also surgeon-captain of the Lancashire Hussars Yeomanry.

The annual Court of Governors of the German Hospital, Dalston, was held on February 2, Mr. A. J. Allen in the chair. In the report the committee stated that the past year had been successful in respect of the large amount of good accomplished by the institution, but that the financial position left much to be desired. The most important event of the year was the completion and opening in July of the Convalescent Home at Hitchin, generously presented to the hospital by Messrs. Fritz and Hans König, and provided with an endowment fund by Baron von Schröder and Baron Bruno von Schröder.

After a protracted sitting the Finsbury Borough Council lately decided, by 29 votes to 16, to abolish its infants' milk depôt and to appoint two female health visitors at a yearly salary of £100 each. In the course of some of the speeches it was mentioned that the depôt has been very little used by mothers of the borough, in which the births are 3,000 yearly. In January of last year there were the names of 70 mothers on the books at the depôt, but there were only 49 in December last. The council has established the depôt for three years, and in each year there was a charge to the rates, the average deficiency being between £400 and £500. It was contended that the depôt has had no material effect in preventing infant mortality. In 1908 the infant mortality for Finsbury was said to be higher than that for 17 boroughs reported to the council.

The Lord Mayor has consented to open the new pathological block of St. Bartholomew's Hospital on Friday, May 7, at 3 o'clock.

Dr. L. Nicholls, of the Colonial Civil Service, has been appointed District Medical Officer, Bacteriologist, and Lecturer on Sanitation in St. Lucia, West Indies.

His many acquaintances among English medical men will be interested to learn that Dr. Schuman Leclercq, Carlsbad, Austria, has been made Chevalier of the French Legion of Honour.

The fifty-ninth annual general meeting of the London Homœopathic Hospital will take place in the Board-room of the Hospital on Friday, March 5, at 3.30 p.m., the Right Hon. the Earl Cawdor presiding.

At a Congregation held on February 11 in the University of Cambridge, Dr. W. H. R. Rivers, M.A., M.D., F.R.C.P., St. John's College, University Lecturer in Experimental Psychology, was appointed to represent the University on the occasion of the celebration in July next of the fiftieth anniversary of the foundation of the Anthropological Society of Paris.

The Annual Court of the Corporation will be held in the Board-room of King's College Hospital on Thursday, February 25, at half-past four o'clock, when will be presented a report of the receipts and disbursements of the past year and of the general state of the hospital, and other special business will be transacted.

The Hon. E. W. B. Portman, presiding at the annual meeting of the Somerset County Hospital at Taunton on February 11, proposed the election as a life governor of Mr. J. C. Bevan, of the Great Western Railway locomotive and carriage departments at Taunton, who during the last twenty years has collected for the hospital £500 in penny subscriptions from his fellow-employés. The proposal was unanimously agreed to.

An effort is being made to arrange special terms for travelling expenses and hotel accommodation for the benefit of those who wish to attend the International Congress of Ophthalmology, which will be held at Naples from March 2 to April 7. Those who wish to attend should make the earliest possible intimation to one of the corresponding members for this country: Mr. Walter H. Jessop, 73 Harley Street, London, W.; Dr. George Mackey, 20 Drumsheugh Gardens, Edinburgh; or Sir Henry Swanzy, 23 Merrion Square, Dublin.

Sir Richard B. Martin, the Treasurer, presided on February 11 at the annual meeting of St. Mark's Hospital for Fistula, City Road. He said that unfortunately the income during the past year was £813 less than the expenditure. The number of in-patients was 523, and the mean residence per patient twenty-one days. Over 6,000 attendances were made by 1,725 out-patients. The nursing accommodation has been increased, and the private wards have been closed, so that now the hospital is wholly free, thus reverting to original conditions. Dr. Swinford Edwards, one of the surgeons, said the result of the year's treatment was considered most satisfactory. There were only five deaths, and the great majority of the patients left the hospital cured. The Samaritan Fund, conducted by the Ladies' Committee, continues to do good work among the poorer patients. Much more support, however, is needed.

Mr. S. A. Ballantyne, M.B., Ch.B.Ed., F.R.C.S.Ed., has been appointed honorary surgeon to the Coventry and Warwickshire Hospital.

The annual general meeting of the Governors of the Cancer Hospital (Free) Fulham Road, S.W., will be held on Wednesday, February 24, 1909, at 4 p.m. precisely.

The annual Court of Governors of the City of London Lying-in Hospital, City Road, London, E.C., will be held at the hospital on Monday, February 22, at 4 o'clock precisely.

Dr. Thompson, medical superintendent of the David Lewis Epileptic Colony, Warford, Cheshire, died on February 16. He served with distinction in the Ashanti expedition for the relief of Coomassie.

The trustees of the bequest of the late Mr. James Dick have allocated more than £311,000 to charities in different parts of Scotland. The Victoria and the Western Infirmaries at Glasgow receive £30,000 each.

The annual general meeting of the Prince of Wales's General Hospital, Tottenham, N., will be held at the hospital on Tuesday, February 23, at 5.15 p.m. Among other business, the annual report and statement of accounts for the year 1908 will be received, and seven members will be elected to serve on the Board of Governors. Subscribers of 10s. and upwards, and donors of £5 and upwards, are entitled to vote at this meeting.

The resignation is announced by Mr. Alban H. G. Doran, F.R.C.S., of his position as senior surgeon to the Samaritan (Free) Hospital for Women, Marylebone Road. Mr. Doran's contributions to the progress and literature of gynæcological surgery during the past three decades are too numerous and well known to require description in detail. In recognition of his services to the hospital, the Committee of Management have decided to hold a complimentary dinner at the Imperial Restaurant on March 9.

At the ordinary meeting of the Council of the College of Surgeons, held on February 1, the President (Mr. Henry Morris) announced that the Prince of Wales had consented to become an Honorary Fellow of the Royal College of Surgeons of England. The votes of the Council were thereupon taken in accordance with the requirements of the charter of the 63rd Vict., and the President declared his Royal Highness to be duly and unanimously elected an Honorary Fellow of the College. The diploma was presented to his Royal Highness, in the presence of the Council, on Monday, February 15, when the Prince and Princess honoured the College with their presence at the delivery of the Hunterian oration by the President.

Princess Victoria of Schleswig-Holstein opened the new wards for children at the Royal Portsmouth, Portsea, and Gosport Hospital on February 3. The Princess declared the wards open, naming them "The Edward and Mary," after Prince Edward and Princess Mary of Wales, and "The Young Ward," after the Chairman of the Building Committee. The hospital was then inspected, and after taking tea in the upper new ward the Princess left for Admiralty House. The hospital dates from 1847, when the foundation-stone was laid by Prince Albert, since which several additions have been made to the building. The new block consists of two main wards, one for boys and one for girls, each containing 20 beds, and forms a further instalment of the original plan for the enlargement and reconstruction of the hospital.

